# Seroprevalence of Hepatitis B and C and HIV Infections and Associated Risk Factors Among Pregnant Women Attending Antenatal Care Unit at Simada Hospital, South Gondar Zone, Northwest Ethiopia

**DOI:** 10.1155/bmri/6895237

**Published:** 2025-01-09

**Authors:** Solomon Tesfaye, Tirngo Abebaw, Endalkachew Bizualem, Daniel Mehabie, Amir Alelign

**Affiliations:** Department of Biology, College of Natural & Computational Sciences, University of Gondar, Gondar, Ethiopia

**Keywords:** Ethiopia, Hepatitis B virus, Hepatitis C virus, HIV, prevalence, risk factors, Simada

## Abstract

Hepatitis and human immunodeficiency virus (HIV) are major public health issues in developing countries, including Ethiopia. These viruses can be transmitted from mother to child during birth or through contact with contaminated blood. In many areas of Ethiopia, viral hepatitis and HIV infections are significant health concerns for pregnant women. However, there is limited information about these issues in various parts of the country, particularly in the Amhara Region. Therefore, this study was aimed at determining the prevalence of Hepatitis B virus (HBV), Hepatitis C virus (HCV), and HIV infections and identifies the associated risk factors among pregnant women attending an antenatal care (ANC) unit at Simada Hospital, Amhara Region, Northwest Ethiopia. A hospital-based cross-sectional study was conducted on 248 pregnant women from January 2021 to March 2021. Five milliliters of venous blood was collected from each study participant, and serum was tested for Hepatitis B surface antigen (HBsAg), anti-HCV, and anti-HIV antibodies using commercially available rapid test kits. A logistic regression analysis was used to identify possible risk factors for the occurrence of hepatitis and HIV infections. The overall seroprevalence of HBV, HCV, and HIV infections was 10 (4%), 5 (2%), and 5 (2%), respectively. Prevalence of coinfection was 1.2% (3/248), 0.4% (1/248), and 0.4% (1/248) for HBV/HCV, HBV/HIV, and HCV/HIV, respectively. History of abortion (adjusted odds ratio (AOR) = 11.028, 95% confidence interval (CI) = 1.671–72.776, *p* value = 0.013) and a history of blood transfusions (AOR = 11.298, 95%CI = 1.066–119.777, *p* value = 0.044) were observed to be significantly associated with an increased risk of HBV infections. However, having multiple sexual partners (AOR = 18.819, 95%CI = 1.074–329.680, *p* value = 0.045) and history of abortion (AOR = 12.550, 95%CI = 1.174–134.202, *p* value = 0.036) were the only significant predictors of HCV and HIV infection, respectively. In the current study, we found an intermediate prevalence of HBV and HCV in pregnant women. Therefore, continuous screening of pregnant women for the stated viral infections should be performed to minimize the transmission of the viruses to their children.

## 1. Background

Inflammation of the liver is generally referred to as hepatitis. It happens as a result of infection with pathogens, primarily the five types of the hepatitis virus (A–E), as well as exposure to drugs, alcohol, toxins, and autoimmune disorders [1]. Regarding viral hepatitis, the Hepatitis B virus (HBV) and Hepatitis C virus (HCV) both have major consequences for public health. For example, HBV and HCV, respectively, caused 296 and 58 million cases of chronic hepatitis infections in 2019. In addition, 1.11 million deaths globally are attributed to HBV and HCV, with HBV accounting for 73.9% (820,000) of these deaths. The primary causes of these deaths are liver cancer and chronic liver disease [2, 3]. As with viral hepatitis, human immunodeficiency virus (HIV) is a serious public health problem globally. For instance, an estimated 37.7 million individuals worldwide were positive for HIV in 2020, including 1.5 million new infections [4].

HIV and hepatitis have been linked to several adverse outcomes during pregnancy, such as intrauterine growth restriction, low birth weight, congenital defects, an increase in perinatal mortality, stillbirths, and spontaneous miscarriages [5–8]. There is a higher risk of morbidity, death, and vertical transmission of HBV, HCV, and HIV from mother to child during pregnancy because these viruses are coendemic, share the same transmission pathway, and coinfect during pregnancy [2–4].

HBV is transmitted horizontally through contact with blood and blood products and vertically from infected mother to child [2]. HCV shares most of the transmission with HBV, but transmission via sexual practices is less common [9]. Studies showed that a history of abortion [10], a history of surgical procedure [11, 12], multiple sexual partners [11, 13], tattooing [14, 15], and blood transfusion [12, 16] were some of the risk factors associated with increased risk of HBV and HCV infection in pregnant women. Regarding HIV infections, multiple sexual partners [17, 18], history of abortion [10, 19], and history of blood transfusion [20] were associated with an increased risk of HIV infection in pregnant women.

Several studies conducted in Ethiopia have shown variations in the prevalence of HIV, HBV, and HCV among pregnant women. For instance, one study at the Attat Hospital in Central Ethiopia found a 4.5% prevalence of HBV and a 1.8% prevalence of HCV [21]. However, another study at the same hospital reported a higher prevalence of HBV at 10.9% [22]. In northeastern Ethiopia, a study at the Borumeda General Hospital found an 8.1% prevalence of HBV and a 3.2% prevalence of HCV [23]. A separate study in the Amhara Region found an overall prevalence of 4.6% for Hepatitis B surface antigen (HBsAg) and 1.6% for anti-HCV antibodies [24]. According to a meta-analysis in Ethiopia, the pooled prevalence of HBV in pregnant women was 4.7% [25].

In studies conducted in Addis Ababa, the Amhara Region, and South–Central Ethiopia, the prevalence of coinfections with HBV and HCV was found to be 1.1%, 1.4%, and 1.8%, respectively [17, 21, 24]. Additionally, the prevalence of coinfection between HCV and HIV was 1.1% in Addis Ababa [17] and 1.8% in South–Central Ethiopia [21].

In the 2014 national antenatal care (ANC) survey, it was found that the prevalence of HIV among pregnant women in Ethiopia was 2.2%. There were regional variations with the highest rates reported in Gambela (7.5%), Harari (6.6%), and Amhara (6.1%) [26]. Studies conducted in rural hospitals in the western and southern parts of Ethiopia reported lower prevalence rates of 1% and 0.2%, respectively, compared to the national average. However, higher rates were found in referral hospitals in the Amhara Regional State (8.68%) [27] and the University of Gondar Referral Hospital (10.33%) [28].

Despite the significant impact on the health of pregnant women and their infants, viral hepatitis, HIV/AIDS, and their coinfections are often overlooked in Ethiopia. There is a lack of routine antenatal screening and intervention strategies for hepatitis, leading to the transmission of the virus from mother to child. Additionally, there is a need for reliable national and subnational epidemiological data on HBV, HCV, HIV, and their coinfections among pregnant women. Therefore, this study is aimed at determining the prevalence of HBV, HCV, and HIV infections and their co-occurrence among pregnant women at Simada Hospital's ANC. The findings will help identify associated risk factors and serve as a basis for developing local and regional control measures.

## 2. Materials and Methods

### 2.1. Study Area Description

Simada is a district in the South Gondar Zone of the Amhara Region in Northwest Ethiopia. The district is bordered by East Este to the west, Lay Gayint to the north, Tach Gayint to the northeast, the Bashilo River to the southeast, and the Nile River to the southwest. The Wanka, a tributary of the Nile, forms part of the border with Este. Additionally, Simada is the largest town in the district.

This district is topographically divided into three sections: 10% highland, 30% mid-highland, and 60% lowland. According to the 2007 Central Statistical Agency (CSA) report, the district had a total population of 228,271; of these, 113,322 were men and 114,949 were women [29]. Most of the population practiced Ethiopian Orthodox Christianity, with 86.92% claiming to be Christians and 13.03% claiming to be Muslims. There is one primary hospital, seven health centers, and 26 health posts servicing the community in the district.

### 2.2. Inclusion and Exclusion Criteria

#### 2.2.1. Inclusion Criteria

Women who had their first ANC follow-up at Simada Hospital during the study period and were willing to participate were included.

#### 2.2.2. Exclusion Criteria

The study excluded women who received their first ANC follow-up at another healthcare facility and were referred to the healthcare facility at the study site. Women who were seriously ill and those unwilling to participate in the study were also excluded.

### 2.3. Study Design and Period

A cross-sectional study was conducted at the ANC unit of Simada Hospital from January 2021 to March 2021.

### 2.4. Sample Size Determination

Using a single population proportion formula, the sample size was estimated to be 248 with a 95% level of confidence, a 3% margin of error, and a 10% nonresponse rate. The prevalence of major blood-borne infections (7.3%) was assumed based on a previous study carried out in Bahir Dar, near the study area [19]. Following the establishment of the sample size, willing pregnant individuals who fulfilled the requirements for inclusion had their blood samples taken.

### 2.5. Data Collection

#### 2.5.1. Data Collections by Questionnaires

A structured questionnaire that was pretested was utilized to gather information about the sociodemographic, behavioral, and clinical factors related to the three viral infections. Data collection was conducted through face-to-face interviews with nurses in the ANC unit of the hospital.

#### 2.5.2. Blood Sample Collection and Test Procedure

An experienced laboratory technologist took a 5-mL venous blood sample from each research participant. Following the manufacturer's instructions, the serum was then separated by centrifugation at 5000 r/min for 15 min. It was then tested for HBsAg and anti-HCV antibodies using one-step HBsAg test strips (Xiamen Boson Biotech Co. Ltd., China) and one-step HCV test strips (Zhejiang Orient Gene Biotech Co. Ltd., China). Retests using the same protocol were performed on samples that tested positive for HCV or HBV; samples that again tested positive for HBsAg and HCVAb were regarded as positive results [21]. The hepatitis kit utilized in the study showed a sensitivity of 99.4% and specificity of 99.5% for HBV, as well as a sensitivity of 99.1% and specificity of 99.2% for HCV. The HIV test was conducted using the Rapid Test Kit algorithm in Ethiopia [30].

#### 2.5.3. Data Quality Assurance

Prior to commencing data collection, the researcher thoroughly reviewed the questionnaire. Initially prepared in English, the questionnaire was later translated into the local language, Amharic, for the participants' ease of understanding. A pilot test was then carried out on 5% of the potential participants who were not part of the actual study, to evaluate the clarity, comprehensibility, and fluency of each question, as well as the time required for completing the questionnaire. Additionally, a 1-day orientation was conducted to ensure that both the facilitators and blood sample collectors had a shared understanding of the tools and methods used for blood sample collection. Standard diagnostic procedures and guidelines were upheld during the blood sample examinations, with confirmatory tests being carried out to verify the reliability of the test protocols.

### 2.6. Data Analysis

The data were manually cleaned, categorized, coded, and entered into SPSS Version 22 software for analysis. Descriptive analysis was used to describe the sociodemographic characteristics and the prevalence of HBV, HCV, and HIV in terms of frequency and percentages. A *p* value less than 0.05 with a two-tailed test was considered statistically significant. Bivariate and multivariate logistic regression analyses were conducted to explore the relationship between the dependent and explanatory variables. Variables with a *p* value of less than 0.25 in the bivariate logistic regression were chosen for further analysis in the multivariate logistic regression [31].

### 2.7. Ethical Considerations

The ethical clearance was secured from the ethical committee of the College of Natural and Computational Science (CNCS), University of Gondar (CNCS/10/638-25-5-2020). The objective of the study was explained to each study participant. The participants were preinformed about the collection of blood samples for HIV, HBV, and HCV screening. Written, agreed-upon, and informed consent for participation was obtained from each study participant. Participants were informed that all the collected data was for academic purposes only. Test results were kept confidential by using unique codes attached to each study participant. Laboratory personnel had access only to the unique codes written on sample-containing test tubes. The researcher had access to both the unique codes and the participants' identities, written in a separate format. All participants were informed of the result, and those with positive result were counseled and linked to the treatment and care units of the health facility.

## 3. Results

### 3.1. Sociodemographic Characteristics of Study Participants

The sociodemographic characteristics of the study participants are summarized in Table 1. Among the surveyed pregnant women, a small percentage (4.8%) was aged 16–20 years. The remaining participants were distributed as follows: 32.7% were aged 21–25, 41.5% were aged 26–30, and 21% were aged 31–45 years.

In terms of religion, the majority of participants (73.8%) identified as Orthodox, followed by Muslims at 22.2%. Of the 248 participants, 38.3% lived in rural areas, while 67.3% resided in urban settings.

Regarding marital status, most participants (70.2%) were married. The remaining individuals were single (14.6%), divorced (12.1%), or widowed (3.2%). Concerning occupation, the majority (45.6%) were housewives, while 22.2% worked in private business, 23.4% were civil servants, and 8.9% were unemployed (see Table 1).

### 3.2. Seroprevalence of HBV, HCV, and HIV Infections

The overall prevalence of HBV, HCV, and HIV infections was 4.0% (10/248), 2% (5/248), and 2% (5/248), respectively. Coinfections with HBV/HCV, HBV/HIV, and HCV/HIV were found in 1.2% (3/248), 0.4% (1/248), and 0.4% (1/248) of pregnant women, respectively (Table 2).

### 3.3. Associated Risk Factors of HBV Infection

Bivariate and multivariate analyses of the different risk factors associated with HBV infection in pregnant women are presented in Table 3. In bivariate logistic regression, having multiple sexual partners, a history of alcohol usage, sharing toothbrushes, having a tattoo, a history of dental procedures, a history of previous abortions, delivery by traditional birth attendants, hospital admissions, a history of blood transfusions, and surgical history were significantly associated with an increased risk of HBV infections. However, in multivariate analysis, a history of previous abortions and a history of blood transfusions remained independent predictors of HBV infection. The likelihood of acquiring HBV infection in pregnant women with a history of previous abortions was observed to be almost 11 times greater (AOR (adjusted odds ratio): 11.028; 95% CI (confidence interval): 1.671, 72.776; *p* value = 0.013) than their counterparts. Likewise, the risk of HBV infection was almost 11 times (AOR 11.298; 95% CI: 1.066, 119.777; *p* value = 0.044) higher in pregnant women who had a history of blood transfusions compared to their counterparts (Table 3).

### 3.4. Associated Risk Factors of HCV Infection

In bivariate logistic regression, having multiple sexual partners, sharing sharp materials, a history of blood transfusion, and a surgical history were associated with HCV seroprevalence. However, the multivariate regression analysis showed that having multiple sexual partners was found to be the only independent predictor of HCV infection. The risk of HCV infection was more than 18 times (AOR: 18.819; 95% CI: 1.074, 329.680; *p* value = 0.045) higher in pregnant women who had more than one sexual partner than in pregnant women who had only one sexual partner (Table 4).

### 3.5. Associated Risk Factors of HIV Infection

In the bivariate logistic analysis, sharing sharp materials, history of dental procedures, history of abortion, and history of blood transfusion were significantly associated with HIV infection. However, only the history of abortion was found to be an independent predictor of HIV infection during multivariate regression analysis. The risk of HIV infection was more than 12 times (AOR: 12.550; 95% CI: 1.174, 134.202; *p* value = 0.036) higher in pregnant women who had a previous history of abortion than in women who had not had abortions (Table 5).

## 4. Discussion

In the present study, the overall prevalence of HBsAg in pregnant women was 4%. The prevalence of HBsAg among pregnant women in this study can be categorized as intermediate (2%–8%) based on World Health Organization (WHO) criteria [2]. The prevalence is also comparable to studies conducted in Bahir Dar City, Northwest Ethiopia (3.8%) [19]; Attat Hospital, South–Central Ethiopia (4.5%) [21]; Dawuro Zone, Southern Ethiopia (3.7%) [32]; and the Democratic Republic of the Congo (3.9%) [33]. However, it is much lower compared to studies conducted in Attat Hospital, South–Central Ethiopia (10.9%) [22]; Borumeda General Hospital, Northeast Ethiopia (8.1%) [23]; Gambella Hospital, Southwest Ethiopia (7.8%) [34]; Yirgalem Hospital, Sidama Region (7.2%) [35]; Yemen (10.8%) [36]; Nigeria (6.78%) [37]; and Cameroon (10.2%) [38]. The observed variations in HBsAg prevalence might result from sample size, location, diagnostic methods, immunization status, cultural practices, and pregnant women's socioeconomic characteristics.

According to this study, 2% of the pregnant women tested positive for HCV, within the 1.5%–3.5% WHO intermediate prevalence category [3]. The prevalence is also comparable with the findings of studies conducted in different parts of the country, such as at Attat Hospital in Southern Ethiopia (1.8%) [21], in hospitals in the Amhara Region (1.6%) [24], and in a meta-analysis study in Ethiopia (1.83%) [39]. This proportion was lower than the HCV prevalence among pregnant women conducted in Western Ethiopia (8.1%) [20], Borumeda General Hospital, Northeast Ethiopia (3.2%) [23], and the Democratic Republic of the Congo (4.1%) [40]. However, it was higher than other studies conducted in Bahir Dar, Northwest Ethiopia (0.6%) [19]; Nigeria (0.8% and 0.5%) [41, 42]; and Southwest China (0.19%) [43]. The study population's diverse socioeconomic background and cultural practices may account for the observed variation in the prevalence of HCV. Furthermore, regional variations in potential risk factors for viral hepatitis, differences in study design, and variations in diagnostic procedures used might have an impact on the previously indicated disparity.

In this study, 2% of the pregnant women were found positive for HIV, which is comparable with the findings of earlier studies conducted in south Ethiopia (2.3% and 1.8%) [44, 45] and the nationwide HIV study in ANC clinics in Ethiopia (2.2%) [26]. However, the present finding was lower than the previous report from Addis Ababa, Ethiopia (4.2%) [14]; Bahir Dar, Northwest Ethiopia (6.6%) [19]; Amhara Regional State (8.68%) [27]; Cameroon (13.1%) [18]; Nigeria (7.3% and 8.5%) [41, 42]; and Tanzania (5.6%) [46]. To the contrary, the HIV prevalence was higher in the present study compared to studies conducted in rural hospitals in Western Ethiopia (1.0%) [20], Southern Ethiopia (0.2%) [44], Southwest China (0.24%) [43], and Iran (0.09%) [47]. The observed difference in HIV prevalence might be attributed to variations in the study populations, variations in the regional and worldwide distribution of HIV, and variations in the diagnostic method.

The prevalence of HBV and HCV coinfection was 1.2% in this study, which is comparable to a study conducted in Addis Ababa, Ethiopia (1.1%) [17]; Attat Hospital, South–Central Ethiopia (1.8%) [21]; and Amhara Region (1.4%) [24]. However, it is higher than studies conducted in Nigeria (0.15%) [37] and the Democratic Republic of the Congo (0.66%) [40]. The observed differences in the frequency of HBV/HCV coinfection could be explained by a variety of factors, including different geographic regions, sample sizes, behavioral and socioeconomic factors, host genetic variations, and diagnostic approaches.

The prevalence of HBV and HIV coinfection was 0.4% in this study, which is comparable to a study conducted in Southwest China (0.24%) [43]; however, this proportion is lower than a study conducted in Addis Ababa, Ethiopia (3.4%) [17]; Bahir Dar City, Northwest Ethiopia (1.3%) [19]; Attat Hospital, South–Central Ethiopia (1.8%) [21]; and Rwanda (4.3%) [48]. In the present study, the prevalence of HCV/HIV coinfection was also 0.4%, which is comparable with a study conducted in Nigeria (0.5%) [42]. However, this proportion is lower than a study conducted in Attat Hospital, South–Central Ethiopia (1.8%) [21], and Addis Ababa, Central Ethiopia (1.1%) [17]. These differences may be explained by the types of research participants employed, their socioeconomic background, cultural norms that encourage the coexistence of the two viral infections, and their degree of knowledge about how diseases spread and how to prevent them.

In the current study, it was found that pregnant women with a history of abortion were almost 11 times more likely to have HBV infection (AOR = 11.028; 95%CI = 1.671, 72.776; *p* = 0.013) compared to women without a history of abortion. This finding is consistent with previous studies conducted in different parts of Ethiopia [14, 19, 21]. Unsafe abortions done outside of healthcare facilities often do not follow proper infection prevention and control measures. This increases the risk of HBV infection among pregnant women who have had abortions. Additionally, unsafe and unwanted sexual practices may lead to abortions, which in turn increase the risk of HBV infections.

The current study also revealed that a history of blood transfusions was a significant independent risk factor for HBV infection. This is consistent with research conducted in Ethiopia and Cameroon [21, 24, 38]. Nonetheless, the results of this investigation contradict those of other research conducted in Ethiopia [14, 15, 17, 23] and outside of Ethiopia [36, 41]. This suggests that HBV can spread through blood transfusions if proper screening is not done. Although blood and blood products have been subjected to HIV, HBV, and HCV testing in Ethiopia [24], molecular techniques are necessary for accurate virus identification to prevent the transmission of HBV during blood transfusion, as ELISA-based HBV screening from blood and blood products may miss the virus during the window period and occult hepatitis.

The study found that having multiple sexual partners was the main risk factor for acquiring HCV infection. This is consistent with a study from Nigeria [13], which also linked having multiple sexual partners with a higher prevalence of HCV. However, in Ethiopia, having multiple sexual partners was not identified as an independent risk factor for HCV transmission [20, 23, 24]. The increased risk of HCV associated with multiple sexual partners in our study might be due to engaging in unprotected sexual activity with different partners, which increases the likelihood of HCV transmission through sexual intercourse.

Similarly, the study found that a history of abortion was the only independent risk factor for HIV infection. This is consistent with findings in pregnant women in Ethiopia [19] and Nigeria [42], where a history of abortion increases the risk of HIV infection. This association may be due to a higher risk of contracting HIV from unsafe abortions, which could result from unprotected sexual activity or unintended pregnancies due to rape.

The current study has some limitations. One of the limitations is that it did not include pregnant women who were unable to access ANC services or those who had follow-ups at the health centers. This could have affected the reported results, leading to a potential underestimation or overestimation of the prevalence of HBV, HCV, and HIV among pregnant women. Additionally, the study's findings were influenced by not using ELISA or molecular approaches for diagnosis, which may have underestimated the prevalence of the three studied viruses.

## 5. Conclusions

The study found that the prevalence of HBV and HCV among pregnant women was moderate. The prevalence of HIV in this study was similar to the 2014 national ANC sentinel surveillance study in Ethiopia. The multivariate logistic regression analysis revealed that having multiple sexual partners and a history of abortions were independent risk factors for HCV and HIV infections, respectively. Meanwhile, having a history of blood transfusions and previous abortions were the main predictors of HBV infections. It is recommended that all pregnant women undergo screening for the hepatitis virus, receive treatment if necessary to reduce their viral levels, and be administered the single-dose Hepatitis B vaccine to prevent transmission to their child.

## Figures and Tables

**Table 1:** Sociodemographic characteristics of pregnant women (*n* = 248) attending the antenatal care unit in Simada Primary Hospital, 2021.

**Variables**	**Category**	**Number**	**Percent**
Age	15–19	12	4.8
	20–25	81	32.7
	26–35	103	41.5
	36–45	52	21

Education level	Illiterate	81	32.7
	Read and write	31	12.5
	Primary level	66	26.6
	Secondary level	50	20.2
	College and above	20	8.1

Religion	Orthodox Christian	183	73.8
	Muslim	55	22.2
	Other religion	10	4.0

Residence	Urban	153	61.7
	Rural	95	38.3

Marital status	Single	36	14.6
	Married	174	70.2
	Divorced	30	12.1
	Widowed	8	3.2

Occupation	Housewife	113	45.6
	Private business	55	22.2
	Civil servant	58	23.4
	Jobless	22	8.9

**Table 2:** Seroprevalence of HBsAg, anti-HCV, and anti-HIV Ab among pregnant women (*n* = 248) attending an antenatal care unit in Simada Primary Hospital, 2021.

**Variables**	**Negative ** **n** ** (%)**	**Positive ** **n** ** (%)**
HBsAg	238 (96.0)	10 (4.0)
HCsAb	243 (98)	5 (2.0)
HIVAb	243 (98.0)	5 (2.0)
HBsAg and HCsAb coinfection	245 (98.8)	3 (1.2)
HBsAg and HIVAb coinfection	247 (99.6)	1 (0.4)
HCsAb and HIVAb coinfection	247 (99.6)	1 (0.4)

**Table 3:** Univariate and multivariate logistic regression analysis of different risk factors and seroprevalence of HBsAg in pregnant women (*n* = 248) attending Simada Hospital Antenatal Care Unit, 2021.

**Variables**	**Negative ** **n** ** (%)**	**Positive ** **n** ** (%)**	**COR (95% CI)**	**p** ** value**	**AOR (95% CI)**	**p** ** value**
*Age*						
15–19	11 (91.7)	1 (8.3)	1.485 (0.141, 15.659)	0.742	NA	
20–25	79 (97.5)	2 (2.5)	0.414 (0.067, 2.563)	0.343	NA	
26–35	99 (96.1)	4 (4.9)	0.660 (0.142, 3.065)	0.596	NA	
36–45	49 (94.2)	3 (5.8)	Ref			
*Education*						
Illiterate	78 (96.1)	3 (3.7)	0.731 (0.072, 7.422)	0.791	NA	
Read and write	30 (96.8)	1 (3.2)	0.633 (0.037, 10.740)	0.752	NA	
Primary level	63 (95.5)	3 (4.5)	0.905 (0.089, 9.212)	0.933	NA	
Secondary level	48 (96.0)	2 (4.0)	0.792 (0.068, 9.253)	0.792	NA	
College and above	19 (95.0)	1 (5.0)	Ref			
*Religion*						
Orthodox Christian	177 (96.7)	6 (3.3)	0.305 (0.033, 2.810)	0.305	NA	
Muslim	52 (94.5)	3 (5.5)	0.519 (0.048, 0.561)	0.519	NA	
Protestant	9 (90.0)	1 (10.0)	Ref			
*Residence*						
Rural	89 (94.7)	5 (5.3)	1.674 (0.472, 5.944)	0.425	NA	
Urban	149 (96.8)	5 (3.2)	Ref			
*Marital status*						
Single	34 (94.4)	2 (5.6)	0.412 (0.033, 5.5.193)	0.412	NA	
Married	168 (97.1)	5 (2.9)	0.207 (0.021, 2.017)	0.207	NA	
Divorced	28 (93.3)	2 (6.7)	0.500 (0.039, 6.336)	0.500	NA	
Widowed	7 (87.5)	1 (12.5)	Ref			
*Occupation*						
Housewife	109 (96.5)	4 (3.5)	0.771 (0.082, 7.243)	0.820	NA	
Private business	54 (98.2)	1 (1.8)	0.389 (0.023, 6.507)	0.511	NA	
Civil servant	54 (93.1)	4 (6.9)	1.556 (0.164, 16.737)	0.700	NA	
Jobless	21 (95.5)	1 (4.5)	Ref			
*Multiple sexual partners*						
Yes	9 (81.8)	2 (18.2)	6.361 (1.178, 34.356)	0.032	1.734 (0.131, 22.920)	0.676
No	229 (96.6)	8 (3.4)	Ref		Ref	
*History of alcohol use*						
Yes	80 (92.0)	7 (8.0)	4.608 (1.161, 18.289)	0.030	1.942 (0.370, 10.184	0.492
No	158 (98.1)	3 (1.9)	Ref		Ref	
*Share toothbrush*						
Yes	6 (75.0)	2 (25.0)	9.667 (1.682, 55.552)	0.011	1.901 (0.100, 36.270)	0.669
No	232 (96.7)	8 (3.3)	Ref		Ref	
*Share sharp material*						
Yes	3 (75)	1 (25.0)	8.704 (0.823, 92.093)	0.072	0.532 (0.004, 69.728)	0.800
No	235 (96.3)	9 (3.7)	Ref			
*History of ear or nose piercing*						
Yes	142 (94.7)	8 (5.3)	2.704 (0.562, 13.011)	0.215	1.317 (0.163, 10.653)	0.796
No	96 (98.0)	2 (2.0)	Ref		Ref	
*Tattooing*						
Yes	86 (92.5)	7 (7.5)	4.124 (1.039, 16.362)	0.044	1.690 (0.266, 10.751)	0.575
No	152 (98.1)	3 (1.9)	Ref		Ref	
*History of dental procedure*						
Yes	33 (89.2)	5 (17.9)	9.348 (1.109, 5.464)	0.035	0.597 (0.603, 5.675)	0.654
No	205 (97.2)	6 (2.8)	Ref		Ref	
*History of abortion*						
Yes	22 (82.1)	4 (14.3)	5.944 (2.517, 34.716)	0.001	11.028 (1.671, 72.776)	0.013
No	214 (97.3)	6 (2.7)	Ref		Ref	
*History of delivery by traditional birth attendants*						
Yes	70 (90.7)	7 (9.1)	5.600 (1.408, 22.281)	0.014	1.726 (0.296, 10.184)	0.547
No	168 (98.2)	3 (1.8)	Ref			
*History of hospital admission*						
Yes	39 (88.6)	5 (11.4)	5.103 (1.410, 18.467)	0.013	2.497 (0.386, 16.159)	0.337
No	199 (97.5)	5 (2.5)	Ref		Ref	
*History of blood transfusion*						
Yes	13 (72.8)	5 (27.8)	17.308 (4.443, 67.423)	0.001	11.298 (1.066, 119.777)	0.044
No	225 (97.8)	5 (2.2)	Ref		Ref	
*Surgical history*						
Yes	16 (84.2)	3 (15.8)	5.946 (1.403, 25.212)	0.016	2.911 (0.372, 24.052)	0.303
No	221 (96.9)	7 (3.1)	Ref		Ref	

Abbreviations: HBsAg, Hepatitis B surface antigen; NA, not applicable.

**Table 4:** Univariate and multivariate logistic regression analysis of different risk factors and seroprevalence of HCsAb in pregnant women (*n* = 248) attending Simada Hospital Antenatal Care Clinic, 2021.

**Variables**	**Negative ** **n** ** (%)**	**Positive ** **n** ** (%)**	**COR (95% CI)**	**p** ** value**	**AOR (95% CI)**	**p** ** value**
*Age*						
15–19	11 (91.7)	1 (8.3)	4.636 (0.269, 79.941)	0.291	NA	
20–25	79 (97.5)	2 (2.5)	1.291 (0.114, 14.609)	0.836	NA	
26–35	102 (99.0)	1 (1.0)	0.500 (0.031, 8.158)	0.627	NA	
36–45	51 (08.1)	1 (1.9)	Ref			
*Education*						
Illiterate	80 (98.8)	1 (1.2)	0.238 (0.014, 3.971)	0.317	NA	
Read and write	30 (96.8)	1 (3.2)	0.623 (0.037, 10.740)	0.633	NA	
Primary level	65 (98.5)	1 (1.5)	0.292 (0.017, 4.897)	0.392	NA	
Secondary level	49 (98.0)	1 (2.0)	0.3889 (0.023, 6.518)	0.388	NA	
College and above	19 (95.0)	1 (5.0)	Ref			
*Religion*						
Orthodox Christian	180 (98.4)	3 (1.6)	0.150 (0.014, 1.589)	0.150	NA	
Muslim	54 (98.2)	1 (1.8)	0.167 (0.010, 2.911)	0.220	NA	
Protestant	9 (90.0)	1 (10.0)	Ref			
*Residence*						
Rural	91 (95.8)	4 (4.2)	6.681 (0.735, 60.701)	0.092	20.483 (0.966, 434.085)	0.053
Urban	152 (99.3)	1 (0.7)	Ref		Ref	
*Multiple sexual partners*						
Yes	9 (81.8)	2 (18.2)	17.333 (2.659, 116.942)	0.003	18.819 (1.074, 329.680)	0.045
No	234 (98.7)	3 (1.3)	Ref		Ref	
*History of alcohol use*						
Yes	84 (96.6)	3 (3.4)	2.839 (0.465, 17.325)	0.258	NA	
No	159 (98.8)	2 (1.2)				
*Share toothbrush*						
Yes	67 (87.5)	1 (12.5)	8.429 (0.831, 85.480)	0.071	0.313 (0.000, 492.394)	0.757
No	237 (98.8)	4 (1.7)	Ref		Ref	
*Share sharp materials*						
Yes	3 (75.0)	1 (25.0)	20.000 (1.693, 236.325)	0.017	1.271 (0.001, 1337.273)	0.946
No	240 (98.4)	4 (1.6)	Ref			
*History of ear or nose piercing*						
Yes	147 (98.0)	3 (2.0)	0.980 (0.161, 5.971)	0.982	NA	
No	96 (98.0)	2 (2.0)	Ref			
*Tattooing*						
Yes	89 (95.7)	4 (4.3)	6.291 (0.762, 62.891)	0.086	27.345 (0.308, 2430.879)	0.148
No	154 (99.4)	1 (0.6)	Ref		Ref	
*History of abortion*						
Yes	26 (92.9)	2 (7.1)	5.564 (0.888, 34.855)	0.064	1.583 (0.088, 28.328)	0.755
No	217 (98.6)	3 (1.4)	Ref		Ref	
*History of blood transfusion*						
Yes	16 (88.9)	2 (11.1)	9.458 (1.473, 60.735)	0.018	6.605 (0.345, 126.315)	0.210
No	227 (98.7)	3 (1.3)	Ref		Ref	
*History of hospital admission*						
Yes	42 (95.5)	2 (4.5)	3.190 (0.517, 19.688)	0.211	1.406 (0.059, 33.283)	0.833
No	201 (98.5)	3 (1.5)				
*Surgical history*						
Yes	17 (89.5)	2 (10.5)	8.863 (1.385, 56.696)	0.021	24.778 (0.504, 1218.278)	0.383
No	226 (98.7)	3 (1.3)	Ref		Ref	

Abbreviations: HCsAb, hepatitis C antibody; NA, not applicable.

**Table 5:** Univariate and multivariate logistic regression analysis of different risk factors and seroprevalence of HIV antibody in pregnant women (*n* = 248) attending Simada Hospital Antenatal Care Clinic, 2021.

**Variables**	**Negative ** **n** ** (%)**	**Positive ** **n** ** (%)**	**COR (95% CI)**	**p** ** value**	**AOR (95% CI)**	**p** ** value**
*Residence*						
Rural	94 (98.9)	1 (1.1)	0.396 (0.044, 3.600)	0.411	NA	
Urban	149 (97.4)	4 (2.6)	Ref			
*Multiple sexual partners*						
Yes	10 (90.9)	1 (9.1)	5.825 (0.595, 57.202)	0.152	0.630 (0.028, 14.099)	0.630
No	232 (98.3)	4 (1.7)	Ref			
*History of alcohol use*						
Yes	86 (98.9)	1 (1.1)	0.456 (0.050, 4.148)	0.456	NA	
No	157 (97.5)	4 (2.5)	Ref			
*Share toothbrush*						
Yes	7 (87.5)	1 (12.5)	8.429 (0.831, 85.486)	0.071	0.534 (0.015, 18.570)	0.729
No	236 (98.3)	4 (1.7)	Ref		
*Share sharp materials*						
Yes	3 (75.0)	1 (25.0)	20.000 (1.692, 236.235)	0.017	12.449 (0.208, 28.768)	0.476
No	240 (98.4)	4 (1.6)	Ref			
*History of ear or nose piercing*						
Yes	146 (97.3)	4 (2.7)	2.658 (0.293, 24.136)	0.385	NA	
No	97 (99.0)	1 (1.0)	Ref			
*Tattooing*						
Yes	89 (95.7)	4 (4.3)	6.921 (0.762, 62.891)	0.086	2.449 (0.208, 28.768)	0.476
No	154 (99.4)	1 (0.6)	Ref			
*History of dental procedure*						
Yes	34 (91.9)	3 (8.1)	9.221 (1.486, 57.224)	0.017	10.126 (0.689, 148.849)	0.091
No	209 (99.1)	2 (0.9)	Ref			
*History of abortion*						
Yes	25 (89.3)	3 (10.7)	13.080 (2.085, 82.066)	0.006	12.550 (1.174, 134.202)	0.036
No	218 (99.1)	2 (0.9)	Ref		Ref	
*History of blood transfusion*						
Yes	16 (88.9)	2 (11.1)	9.458 (1.473, 60.735)	0.018	3.381 (0.283, 40.463)	0.336
No	227 (98.7)	3 (1.3)				
*Hospital admission*						
Yes	43 (97.70	1 (2.3)	1.163 (0.127, 10.663)	0.894	NA	
No	200 (98.0)	4 (2.0)				
*Surgical history*						
Yes	18 (94.7)	1 (5.3)	3.125 (0.332, 29.451)	0.319	NA	
No	225 (98.3)	4 (1.7)	Ref			

Abbreviations: HIV, human immunodeficiency virus; NA, not applicable.

## Data Availability

The corresponding author can share data when reasonable requests emerge.

## Nomenclature

AOR: adjusted odds ratio
ANC: antenatal care
HBsAg: Hepatitis B surface antigen
HBV: Hepatitis B virus
Anti-HCV: anti-Hepatitis C virus antibody
HCB: Hepatitis C virus
CI: confidence interval
COR: crude odds ratio
HIV: human immunodeficiency virus
